# A Study of a Decade of Road Traffic Accidents in Benghazi - Libya: 2001 to 2010

**DOI:** 10.1371/journal.pone.0040454

**Published:** 2012-07-11

**Authors:** Zuhir Bodalal, Riyad Bendardaf, Mohammed Ambarek

**Affiliations:** 1 Faculty of Medicine, Libyan International Medical University, Benghazi, Libya; 2 Department of Oncology and Hematology, Benghazi Medical Center, Benghazi, Libya; 3 Consultant Orthopedic Surgeon, Benghazi, Libya; Tehran University of Medical Sciences, (Islamic Republic of Iran)

## Abstract

This paper aims to observe and to study the trends of road traffic accidents (RTA’s) for the past ten years in Benghazi – Libya. A retrospective analysis was done using the patient records of Al-Jalaa hospital (the main trauma center in Benghazi) from over 21,753 RTA cases. The annual data were compared to each other and changes of trends were observed. RTA’s represented an increasing percentage of Al-Jalaa’s case load across the years. Around 41% of these cases needed to undergo surgery. The younger age group (20–29 years of age) formed the majority of cases while there was a trend towards an increasing average age of patients involved in an accident. Male patients were found to be younger than their female counterparts. Males comprised 81.5% while females formed 18.5% of RTA patients. In terms of inpatient duration, most patients stayed in the hospital for less than 1 week. Vehicle occupants (drivers and passengers) were admitted more often than pedestrians. There was a trend across the years towards an increased involvement of vehicle occupants and decrease in the proportion of pedestrians that had to be hospitalized. Additionally, there was a decrease in the fatalities of pedestrians. Overall, most RTA patients were discharged and made to follow-up in outpatient clinics however there was a startling trend towards increased LAMA and absconded patients. There were both encouraging findings as well as points that needed further emphasis and action. Public education, life support training and diversification of transport (*apart from the use of the roads*) should be looked into, as possible means of improving the current situation.

## Introduction

Road traffic accidents are a major source of public health concern in Libya. This paper covers the extent of this concern over the past decade for the city of Benghazi – Libya. According to the WHO, Libya has the third highest per capita of fatal RTA’s in the world with a value of *40.5/100,000* inhabitants per year [1]. Only Eritrea and the Cook Islands have higher values in terms of per capita traffic accident deaths.

Libya is a coastal North African nation and is classified under the Eastern Mediterranean Regional Office (EMRO). According to data in 2007, its population was 6.1 million. It is a middle income nation (per capita $9010) with over 1.8 million registered vehicles. [1].

Libya has made progress with regards to public health and sanitation. As a result of the development, many diseases that were endemic began to decrease in incidence and prevalence. This gave way to a comparative increase in those being affected by injuries, most commonly road traffic accidents. [2].

This problem is faced by many countries in the region that have been developing economically. As the standard of living increases, more cars are on the road and as a corollary, there are more accidents. Qatar in particular is suffering from a high incidence of RTA’s and recently motor vehicle collisions were found to be the leading cause of death in the country. [3].

The economic impact has also important since RTA’S result in economic losses equivalent to 1 - 2% of the gross national product of most countries. [4] This study also covers that aspect in terms of hospital accommodations expenses.

Unfortunately, despite the extent of this issue and the large number of people affected, not enough research in being made in relation to motor vehicle collisions in Libya. Therefore, relying on the given data, an outline will be presented on the current status of this problem.

This paper aims at providing much needed data on the current status of road traffic accidents in Libya. Moreover, an effort was made to highlight the various parameters that played a role in road traffic accident patients.

## Materials and Methods

### (i) Ethical Approval

This study was approved by the Libyan International Medical University Ethical Committee to have met international biomedical ethics guidelines. Moreover, the acquisition of data was supervised by both the University and the Biostatistics Department at Al-Jalaa hospital.

### (ii) Data

Data was used from the Biostatistics Department of Al-Jalaa hospital in Benghazi – Libya. This hospital serves the eastern half of the country. Al-Jalaa hospital receives the overwhelming majority of RTA’s in the Eastern part of Libya and is therefore able to serve as a good indicator of this condition for the whole region, if not all of Libya.

The data obtained spanned ten years (2001–2010) and each year was studied separately. Various parameters were observed and recorded for each year and different conclusions were made either through generalization or through trends.

### (iii) Statistical Methods

An Excel based model was designed that spanned the collected data and basic statistical procedures were performed. These included means, standard deviations, percentage calculation, and table and chart design.

The admission data was taken for all the cases admitted to all the departments of the hospital. Furthermore, the data was filtered to include only those cases that were admitted because of RTA’s, regardless of the role (pedestrian, driver or passenger) or the type of vehicle (4-wheeler or 2-wheeler).

The parameters under observation were age, gender, nationality, role in the accident, duration of hospital stay, operations, method of discharge and fatalities etc. In addition, the results obtained from this data were compared to the same figures from previous years (pre-2000) for Libya.

## Results

For the purpose of convenience, the results will be presented under various headings. The headings will be based on various parameters previously mentioned. The findings will be presented for each individual year and finally a generalization is made to provide the overall finding for that particular variable. In addition, tables and charts will be utilized.

### (i) Magnitude of the problem

Al-Jalaa hospital receives a large portion of patients from Benghazi and the other cities of Libya’s Eastern region. On average, it admits 16,850 cases annually and of those cases 12.9% are due to road traffic accidents. In the year 2010, RTA admissions were only exceeded by patients who had suffered from falls (from a height or otherwise) who formed 17.1% (n = 3276).

Across the past ten years, Al-Jalaa witnessed 21,753 RTA patients, which are approximately six patients per day. However, if we were to look at each year individually (as in **Table 1**), it would be seen that the year 2010 witnessed 7.26 patients a day.

**Table 1:** Display of the total number of cases per year/day as well as the contribution of road traffic accidents to the total admissions at Al-Jalaa hospital.

Year	No. of RTA cases	No. of RTA cases per day	% of total admission
2001	1613	4.42	10.1%
2002	1681	4.60	11.5%
2003	1940	5.31	12.0%
2004	2054	5.62	12.8%
2005	2180	5.97	13.3%
2006	2186	5.98	13.6%
2007	2372	6.49	13.7%
2008	2491	6.82	13.8%
2009	2583	7.07	13.8%
2010	2653	7.26	13.8%
**Overall**	**21753**	**5.96**	**12.9%**

Additionally, a significant number of operations are performed for these patients, 41.4% (n = 4,824) of whom required it (for patients from the period of 2001–2006). **Table (2** **)** shows the trend towards the performing of surgery for RTA patients at Al-Jalaa hospital.

**Table 2:** The trend towards performing surgery on road traffic accident patients from the year 2001–2006 at Al-Jalaa hospital.

Year	Not Operated (n|%)	Operated (n|%)
2001	1232 (76.4%)	381 (23.6%)
2002	1145 (68.1%)	536 (31.9%)
2003	1149 (59.2%)	791 (40.8%)
2004	1305 (63.5%)	749 (36.5%)
2005	1443 (66.2%)	737 (33.8%)
2006	1408 (64.4%)	778 (35.6%)
**Overall**	**6836 (58.6%)**	**4824 (41.4%)**

### (ii) Nationality

The majority of patients (89.9% n = 19,546) admitted to Al-Jalaa hospital were Libyans.

### (iii) Age

The age distribution of the patients admitted to the hospital displayed a wide range– the youngest child being 1 month old and the oldest being 95 years. Interestingly, there is a very prominent peak that occurs in the 20–29 year old (*yo*) age group. This age group formed 28.9% (n = 6285) of all the traffic accident patients. The number of patients admitted to the hospital for that particular age group is almost twice that of the 10–19 age group (n = 3346) and around 1.5 times more than the 30–39 age group (n = 4278). The most common age for admission due to an RTA is 25 years and the importance of this age group is that it has the maximum productivity. Furthermore the average age of patients admitted to the hospital (as a result of RTA’s) is within that age group (as shown in **Table (3** **)**).

**Table 3:** Average age and standard deviation (SD) of patients admitted as a result of traffic accidents.

	Avg. Age	SD
2001	28.63	16.76
2002	28.45	16.91
2003	29.16	16.85
2004	29.74	16.63
2005	29.94	16.94
2006	29.32	16.32
2007	29.48	16.63
2008	29.76	16.39
2009	30.35	16.20
2010	30.86	16.91

Fifteen thousand, nine hundred and twenty two (15,922) patients (26.7%) were classified as adults whereas 5803 patients (73.3%) were 19 years of age and under.

One point worth mentioning in this calculation of the distribution of age is that in 28 cases (out of 21,753) the age was not provided and hence they were not included in the calculation of parameters related to age. However, all the other data was present and therefore were included in the other parameters.

### (iv) Gender

Of the 21,753 patients spanning the different years observed in this study, 17,726 (81.5%) were male and 4027 (18.5%) were female. The average age of admitted females was 30.5 (±18.7) whereas the average age of males was 29.4 (±16.3). The different distributions of gender per year (along with various other parameters) are given in **Table (4** **).**

**Table 4:** Trends of roles, duration spent, age distribution and method of discharge for RTA’s from the year 2001–2010 with an overall summary.

	2001		2002		2003		2004		2005		2006		2007		2008		2009		2010		Overall	
**Age Distribution (n | %)**																						
0–9 yo	186	11.6%	226	13.5%	213	11.0%	232	11.3%	241	11.1%	242	11.1%	285	12.0%	279	11.2%	277	10.7%	276	10.4%	**2457**	**11.3%**
10–19 yo	289	18.0%	276	16.5%	340	17.6%	308	15.0%	314	14.4%	361	16.5%	380	16.0%	359	14.4%	379	14.7%	340	12.8%	**3346**	**15.4%**
20–29 yo	471	29.3%	498	29.7%	573	29.6%	604	29.5%	647	29.7%	615	28.1%	647	27.3%	706	28.4%	744	28.8%	781	29.4%	**6285**	**28.9%**
30–39 yo	280	17.4%	305	18.2%	351	18.1%	387	18.9%	445	20.5%	458	21.0%	470	19.8%	502	20.2%	535	20.7%	544	20.5%	**4278**	**19.7%**
40–49 yo	140	8.7%	122	7.3%	165	8.5%	204	10.0%	206	9.5%	208	9.5%	223	9.4%	263	10.6%	274	10.6%	311	11.7%	**2116**	**9.7%**
50–59 yo	130	8.1%	110	6.6%	145	7.5%	166	8.1%	143	6.6%	140	6.4%	188	7.9%	187	7.5%	175	6.8%	189	7.1%	**1573**	**7.2%**
60–69 yo	66	4.1%	99	5.9%	100	5.2%	101	4.9%	116	5.3%	118	5.4%	137	5.8%	134	5.4%	133	5.2%	131	4.9%	**1135**	**5.2%**
70–79 yo	33	2.1%	36	2.1%	37	1.9%	40	2.0%	52	2.4%	40	1.8%	39	1.6%	48	1.9%	53	2.0%	60	2.3%	**438**	**2.0%**
80 and up	10	0.6%	3	0.2%	12	0.6%	7	0.3%	11	0.5%	4	0.2%	4	0.2%	12	0.5%	13	0.5%	21	0.8%	**97**	**0.4%**
**Duration (n | %)**																						
No Stay	64	4.0%	78	4.6%	119	6.2%	142	6.9%	167	7.7%	169	7.7%	178	7.5%	185	7.4%	190	7.4%	194	7.3%	**1487**	**6.8%**
1–7 days	874	54.2%	981	58.4%	1155	59.5%	1235	60.1%	1323	60.7%	1344	61.5%	1514	63.8%	1619	65.0%	1694	65.6%	1755	66.2%	**13492**	**62.0%**
8–14 days	267	16.6%	273	16.2%	313	16.1%	331	16.1%	350	16.1%	360	16.5%	375	15.8%	386	15.5%	396	15.3%	402	15.2%	**3452**	**15.9%**
15–29 days	203	12.6%	215	12.8%	233	12.0%	239	11.6%	245	11.2%	218	10.0%	213	9.0%	211	8.5%	212	8.2%	211	8.0%	**2199**	**10.1%**
30+ days	108	6.7%	100	5.9%	100	5.2%	98	4.8%	95	4.4%	95	4.3%	92	3.9%	91	3.7%	92	3.5%	91	3.4%	**962**	**4.4%**
Unspec.	97	6.0%	34	2.0%	20	1.0%	10	0.5%	0	0.0%	0	0.0%	0	0.0%	0	0.0%	0	0.0%	0	0.0%	**161**	**0.7%**
**Role (n | %)**																						
Pedestrian	558	34.6%	583	34.7%	563	29.0%	N/A	N/A	647	29.7%	691	31.6%	732	30.8%	749	30.1%	738	28.6%	677	25.5%	**5938**	**30.14%**
Driver	376	23.3%	416	24.7%	418	21.5%	N/A	N/A	704	32.3%	586	26.8%	659	27.8%	716	28.7%	792	30.6%	915	34.5%	**5582**	**28.33%**
Passenger	538	33.4%	653	38.8%	657	33.9%	N/A	N/A	812	37.2%	866	39.6%	934	39.4%	975	39.2%	999	38.7%	1002	37.8%	**7436**	**37.74%**
Motorcycle	20	1.2%	7	0.4%	21	1.1%	N/A	N/A	17	0.8%	18	0.8%	22	0.9%	25	1.0%	31	1.2%	41	1.5%	**202**	**1.03%**
Bicycle	26	1.6%	22	1.3%	25	1.3%	N/A	N/A	0	0.0%	25	1.1%	26	1.1%	26	1.0%	24	0.9%	18	0.7%	**192**	**0.97%**
Unspec.	95	5.9%	0	0.0%	256	13.2%	N/A	N/A	0	0.0%	0	0.0%	0	0.0%	0	0.0%	0	0.0%	0	0.0%	**351**	**1.78%**
**Method of Discharge (n | %)**																						
Transferred to another hospital	11	0.68%	20	1.19%	16	0.82%	18	0.88%	21	0.96%	27	1.24%	36	1.52%	41	1.65%	44	1.70%	47	1.77%	**281**	**1.3%**
LAMA	48	2.98%	59	3.51%	89	4.59%	90	4.38%	132	6.06%	180	8.23%	206	8.69%	222	8.91%	234	9.06%	243	9.16%	**1503**	**6.9%**
Absconded	33	2.05%	22	1.31%	47	2.42%	56	2.73%	60	2.75%	67	3.06%	75	3.16%	80	3.21%	84	3.25%	87	3.28%	**611**	**2.8%**
Discharged to attend OPD	1313	81.40%	1455	86.56%	1663	85.72%	1778	86.56%	1802	82.66%	1732	79.23%	1899	80.09%	2005	80.49%	2084	80.68%	2146	80.89%	**17877**	**82.2%**
Expired	107	6.63%	90	5.35%	107	5.52%	108	5.26%	156	7.16%	177	8.10%	147	6.20%	131	5.26%	124	4.80%	115	4.33%	**1262**	**5.8%**
Unspecified	101	6.26%	35	2.08%	6	0.31%	1	0.05%	0	0.00%	0	0.00%	0	0.00%	1	0.04%	1	0.04%	1	0.04%	**146**	**0.7%**
Refuse Adm.	0	0.00%	0	0.00%	12	0.62%	3	0.15%	9	0.41%	3	0.14%	8	0.34%	11	0.44%	12	0.46%	14	0.53%	**72**	**0.3%**

(yo =  years old, Refuse Adm. =  Refuse Admission, OPD =  Outpatient Department, LAMA =  Left against medical advice).

### (v) Length of hospital stay and its costs

The number of RTA cases admitted to Al-Jalaa hospital increases every year. On average RTA’s contributed to 12.9% of the total admissions in the past ten years. This contribution is proportionately increasing every year whereas in 2010 around 13.8% of the patients admitted (n = 2,653) were as a result of motor vehicle collisions. The number of days that patients spent in the hospital was observed. Interestingly, 6.8% (n  = 1487) of patients were discharged in less than 24 hours. However the vast majority of patients (62% n = 13,492) spent from 1 day to a full week before being discharged. **Table (5** **)** depicts the duration spent by the patients as a result of RTA’s for the past ten years.

**Table 5:** The duration spent by RTA patients who were brought to Al-Jalaa hospital.

Duration	No. of patients	Percentage
No Stay	1487	6.8%
1–7 d	13492	62.0%
8–14 d	3452	15.9%
15–29 d	2199	10.1%
30+d	962	4.4%
Unspecified	161	0.7%
**Overall**	**21753**	**100%**

According to the hospital biostatistics department, the cost for spending a day in Al-Jalaa is 250 LD ($192 US). It was found that, across the duration under study (from the year 2001 to 2010), RTA patients spent 176,214 days in the hospital. When calculated, that comes to a cost of over 44 million Libyan dinars (33.8 million US dollars) in hospital accommodation costs alone (further details are shown in **Table 6**).

**Table 6:** The total number of days spent by RTA patients at Al-Jalaa hospital and the cost for accommodating them in LD (Libyan Dinars).

Year	Days spent	Cost (LD)
2001	16,790	4,197,500
2002	17,529	4,382,250
2003	17,454	4,363,500
2004	17,436	4,359,000
2005	17,379	4,344,750
2006	17,417	4,354,250
2007	17,824	4,456,000
2008	18,027	4,506,750
2009	18,128	4,532,000
2010	18,230	4,557,500
**Overall**	**176,214**	**44,053,500**

### (vi) Role

In this study, the roles of the victims in the road traffic accident were divided into five categories; pedestrian, passenger, driver, bicycle and motorcycle. **Table (4** **)** displays the distribution of victims among the various roles.

As can be seen, the majority of cases are vehicle occupants (driver and passengers) forming approximately 66% (n = 13,108) of the total number of patients.

Drivers, in particular, are being increasingly injured where they constitute 28.3% (n = 5,582) of the RTA patients admitted to the hospital. In addition, 23.1% (n = 266) of the fatalities are drivers. Also, 1.6% (n = 87) of the drivers admitted were underage (≤17 years old).

Passengers formed the bulk of the patients admitted to the hospital for injuries (37.7% n = 7,436). Understandably, there was a male predilection (as with all other subsets), however, this time only 68.4% (n = 5,086) were male. To add to that, 18.1% (n = 1349) of the passengers were minors and hence more vulnerable than adults. Vehicle occupants (drivers and passengers) were mostly male (81% n = 10,544).

The most interesting figures come from the pedestrian subset. They constitute 30.1% (n = 5,948) of the total number of RTA patients admitted to hospital.

Nearly half (45.9% n = 2,647) of the pedestrians were underage.

### (vii) Method of Discharge

The methods of discharge were categorized as: Transferred to another hospital, Discharge for follow-up in the OPD (out-patient department), LAMA (leave against medical advice), Absconded, Expired, Refused admission and Unspecified. Due to documentation difficulties, some patients’ method of discharge was left absent and hence recorded under unspecified.

The overwhelming majority of patients (82.2% n = 17,877) were discharged and continued for follow-up in the OPD.

Patients who left against medical advice or absconded (ran away) together formed 9.7% (n = 2,114).

### (viii) Fatality

The overall number of patients who died as a result of RTA’s was 1262 patients (5.8%). The general trend shows that the percentage of fatal car accidents has decreased from a value of 6.6% in 2001 to just above 4.3% in 2010. For every patient that died as a result of a car crash, 16.2 patients were injured either slightly or seriously.

Despite being 81.5% of the total number of accident patients, males form 84.3% (n = 1,064) of the deaths (perhaps indicating a predilection towards males).

The age group that showed the largest number of deaths was 20–29 years old (21.6% n = 272), which is logical, given their numerical superiority in comparison to the other age groups. However, surprisingly the 30–39 year old age group showed a similar proportion in terms of death (20.7% n = 260) despite having half the number of patients in that age group as compared to the one before it.

It is worth mentioning that while passengers form the majority of RTA injuries, pedestrians have the greatest risk of being in a fatal car crash. The number of deaths that occurred as a result of road traffic accidents was 1264 deaths and of that number, pedestrians, drivers and passengers formed 38.4%, 23.1% and 35.5% respectively.

In addition, RTA’s are the cause of 25–30% of the total deaths that occur in Al-Jalaa hospital. This number has been on the rise and **Table (7** **)** shows this parameter and its trend across the years.

**Table 7:** The proportion of deaths as a result of road traffic accidents from the total number of deaths that occur at Al-Jalaa hospital.

Year	Deaths
2001	22.81%
2002	28.85%
2003	29.89%
2004	32.53%
2005	26.26%
2006	27.92%
2007	31.28%
2008	32.96%
2009	33.80%
2010	34.64%

## Discussion

There is a clear and visible increase in the number of motor vehicle collisions in Benghazi [3], [5] and proportionately more people are taken to Al-Jalaa as a result of car accidents. Also, when the number of RTA admissions per day was calculated, it was found to be sharply raised from previous calculations (3.4 RTA patients per day). [6].

The predominance of Libyan national patients as opposed to foreign citizens is understandable given the fact that the majority of the population is Libyan, however further research is necessary to appropriately comment on the matter.

As can be seen from table (4), there is a general trend towards an increase in age across the past decade. The trend is seen not only in the average age but also in the general distribution of age. The proportion of patients aged 30 and above is increasing. This may be seen as a positive effect of enforcement of underage driving laws.

In societies where women’s mobility is traditionally restricted, men may spend substantially more time in moving vehicles than women, and among all groups other than among the small economic elite, men are more likely to own cars than women. Men are also more likely to be employed as drivers and mechanics in cars and trucks, including drivers of long-haul vehicles which may mean spending several days and nights in the vehicle. Males, therefore, have a higher exposure to the risk of traffic injuries. [7].

Libya’s gender proportion (*among RTA patients*) shows a great difference as compared to the statistics from other nations such as Australia with 66% being male [8] or Turkey with 68% [9]. However, the figures from Libya come near the ratio found in other developing countries such as India with 83% of the victims of RTA’s being male [10] or 86% in Pakistan [11]. The bias observed towards male involvement in car accidents can be explained by the fact that, in the Libyan society, females tend to participate less in outdoor activities. [12].

In concurrence with the increased number of cars (motorization) [13], the general trend across the years is a decrease from pedestrian injuries and an increase in vehicle occupant injuries. [14].

The overwhelming majority of drivers are male (97.8% n = 5458) which differs from previous research spanning till the year 2000 that stated that 91% of the drivers injured are male. [6].

This shows a decrease from the 1.8% that was previously calculated for Libyan RTA’s. [6] This decrease in the incidence of underage drivers most likely derives from the enforcement of underage driving laws in Benghazi.

The ratio of male to female passengers was 2.2∶1 as opposed to the previous 1.9∶1 [6]. Taking into consideration the increased proportion of women in this category, women are far more likely to be involved in a motor vehicle collision as a passenger than any other role.

It was observed that vehicle occupants were mostly male. This corroborates with previous values calculated for Libya in the earlier years. [15] [6] Another recent study that studied the gender distribution between European vehicle occupants found males to form 67%. [16].

The proportion of pedestrians being involved falls across the years as more and more vehicle occupants are being affected. This percentage is much lower when compared to values found in other researches for Africa (40% pedestrian) and the Middle East (50% pedestrian) and it seems to be mid-way between the values of Africa and the US/EU (20% being pedestrians). [17].

In the Libyan scenario, nearly half the pedestrians were underage. This shows contrast with previous works that had placed the value at 30% [18]. One possible explanation for the over-representation of minors among pedestrian injuries and fatalities would be that the young and old both spend more time walking on the streets to reach their destination as compared to middle aged people.

The overwhelming majority of patients were discharged and made to follow up in the OPD. This may stem from the general policy of the hospital to stabilize patients and refer them to the OPD in order to circumvent any economic burden on the hospital.

Across the years, there has been an alarming trend towards increasing the number of patients who leave by this method.

In 2010, nearly half of the patients admitted to the Centre for the Rehabilitation of the Disabled in Benghazi were as a result of road traffic accidents. [19].

Previous research in the EMRO (Eastern Mediterranean Region Office) had placed the distribution of fatalities at 73% male and 27% female. [20].

In accordance with previous works, the percentage of underage deaths was over one fifth (20.1% n = 253). [23] Although one striking difference was that in the reference used, the term “underage” was defined as below 15 years of age, however for the purposes of this study, underage means a person below the age of 18 (a minor).

Previously, it was thought that in Libya, 46% of deaths were pedestrians [6]. That figure has now decreased to form 38.4% (n = 444) and that is due to less people walking around or a credit to the efforts made to help decrease pedestrian injuries, either in the form of educating the population on the proper methods of crossing streets or setting up road-crossings. [21] Male pedestrian injuries have increased in the past ten years and now form 80.6% (up from a previous 66% [6]). Looking at countries from the region, this value is similar to the 44% male pedestrian injuries found in a hospital in Tehran. [22].

### Conclusions

This study has shown the data recorded for road traffic accidents in the past ten years for the largest hospital in Benghazi. Given its size and the case load that it receives from all the different parts of the east, a broader trend can be made regarding the status of the motor vehicle collision problem that the Libyan society is facing. On various aspects, there have been both encouraging findings and points that may need further enforcement.

As we have seen, the proportion of underage drivers has decreased across the years. Underage drivers are generally far more likely to be involved in a road traffic incident and harm not only themselves but others around them. Similarly, the average age of people injured/killed as a result of RTA’s has increased meaning that there are fewer younger people being affected.

As a result of both educational campaigns and traffic law enforcement, the proportion of pedestrians that are either injured or killed has decreased. Another factor that may have helped in this decreased proportion would be the increased vehicle occupant involvement as a result of increased motorization in our society.

In that light, some recommendations that can be made are:

- There is urgent need for education of the public through the use of news media and television programmes. [24] They should encourage the wearing of seat belts, the enforcement of all the traffic laws and subsequently punishing all those who transgress these laws. Among these laws that need to be enforced would be better driver training programs and implementation of license and registration laws.
- Life support training given to the public would be very useful considering that BLS could be given to patients while the ambulance is en route. ATLS training given to professionals in the casualty department was emphasized in previous works as being an important point that needs work. [25], [26]
- Detailed analysis of the causes of road traffic accidents in Libya in general and eastern Libya in particular is required. Armed with this information, preventive measures can be taken that would save countless lives and millions of dollars for the national economy.
- One of the major causes for RTA’s is that the only viable means of mass transport across the nation is through the road. This puts pressures on the traffic and increases the risk of an accident occurring. Efforts should be made to diversify the means of transport of freight goods and people.
